# Supplementary material for: Simulation of slip transients and earthquakes in finite thickness shear zones with a plastic formulation
Source: Nat Commun. 2018 Sep 25;9:3893. doi: 10.1038/s41467-018-06390-z (PMC6156592; doi:10.1038/s41467-018-06390-z)
Supplement: Supplementary file 1 — Supplementary Information [file 41467_2018_6390_MOESM1_ESM.pdf]

Supplementary information for:

# Simulation of slip transient and earthquakes in finite thickness shear zones with a plastic formulation

By X. Tong and L. Lavier

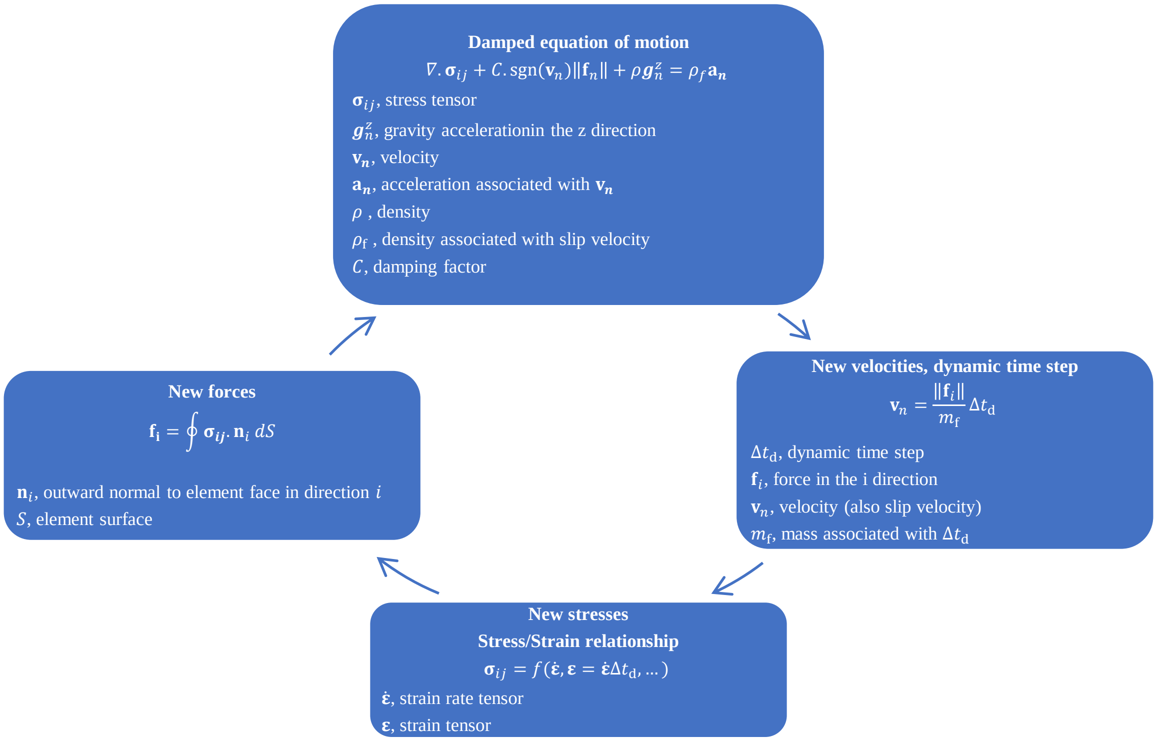


**Supplementary Figure 1.** This figure illustrates the FLAC (Fast Lagrangian Analysis of Continua) algorithm for each time step. Strain rate, strain, and stress are tensor quantities (element-wise). Force, acceleration, and velocity are vectors (node-wise). Our application of the rate-dependent friction formulation occurs when new stresses are estimated. Supplementary Figure 2 describe the evolution of the plastic formulation of rate and state. We calculate the adaptive time step after the velocity has been updated. See also method section.

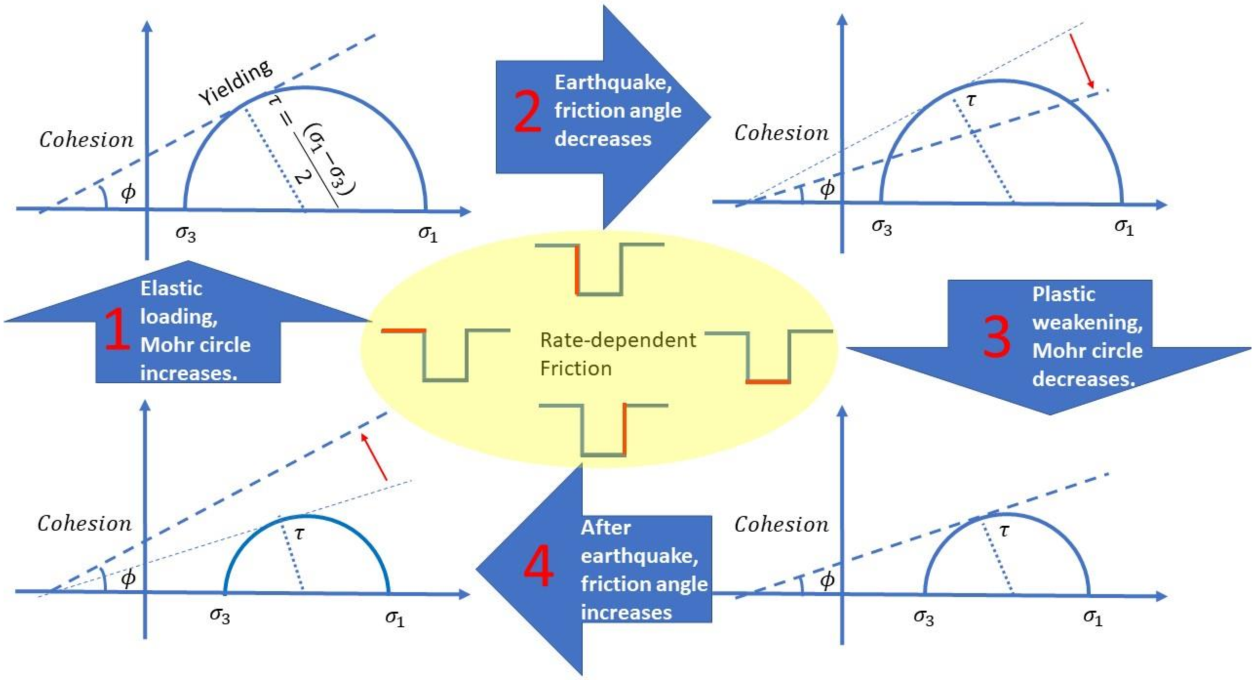


**Supplementary Figure 2.** This figure illustrates the evolution of our plastic formulation of rate dependent friction. There are four stages: 1) Interseismic: if the state of stresses of an element is inside the failure envelope, the stresses increase elastically until yielding. 2) Seismic: when an earthquake happens, the friction angle decreases as slip rate increases. 3) Seismic: the element is weakened plastically, a new failure envelope is defined and the state of stresses decreases until the Mohr circle returns inside the new failure envelope. During this period plastic strain or damage is accumulated and generates a delayed drop in shear stress. 4) Interseismic: after an earthquake, the friction angle increases as the slip velocity decreases. The element is once again in stage 1 during which the stress state increases elastically.

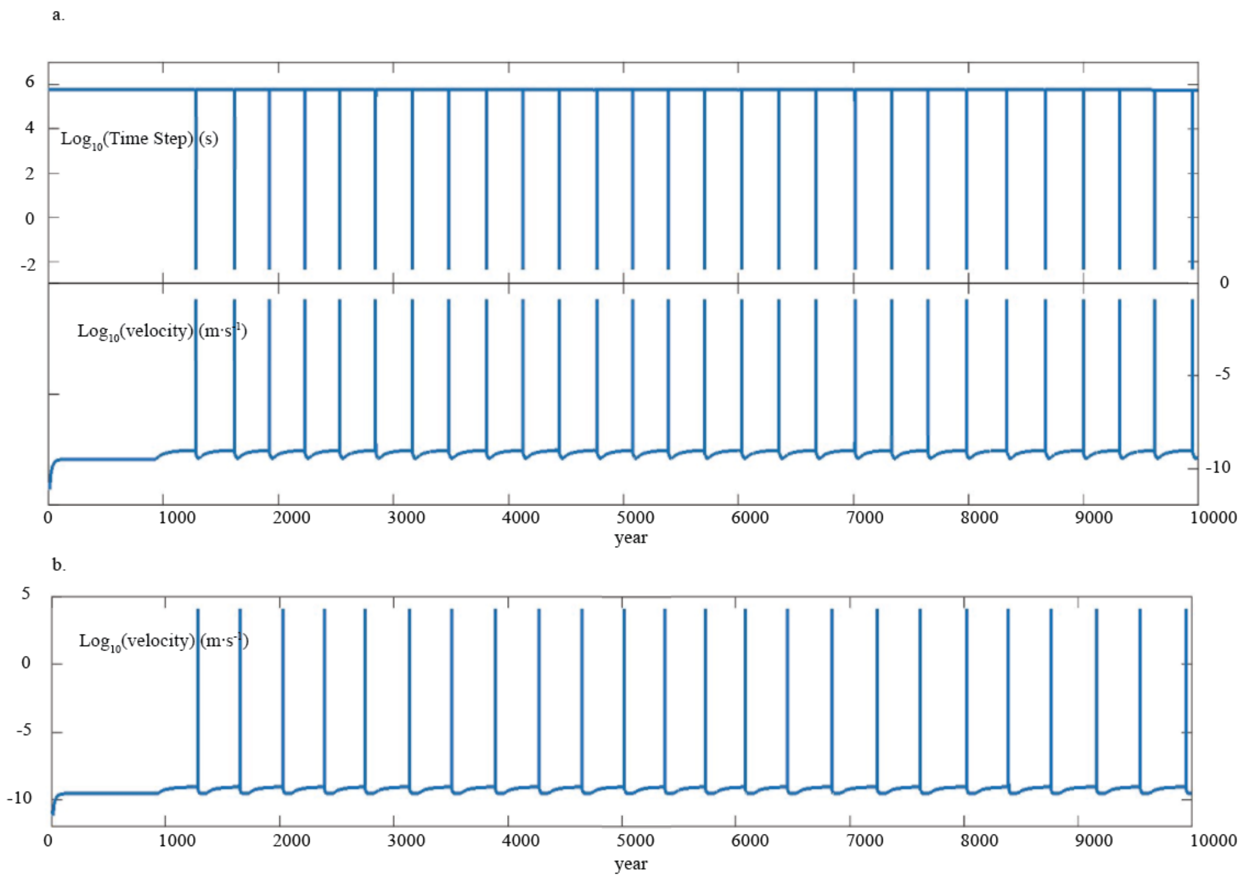


**Supplementary Figure 3.** a) Upper part plots the adaptive time step in log scale, and lower part plots the velocity evolution over 10,000 years. The adaptive time step drops to a fraction of second during a seismic event while it reaches to about 10 days during interseismic period. b) Shows the velocity evolution when the force balance was solved quasi-dynamically. Slip speeds reach to shear wave velocity (~ 4900 $m \cdot s^{-1}$) during earthquakes.

**Supplementary Table 1.** Input parameters for this study.

| Density | Bulk Modulus | Shear Modulus | Cohesion |
|---|---|---|---|
| 2800 $kg\cdot m^{-3}$ | 123 GPa | 67 GPa | 4 MPa |
| **Dilation Angle** | **Reference Friction Coefficient** | **Reference Slip Velocity** | **Tectonic Loading Rate** |
| 0 | 0.6 | $10^{-6}$ $m\cdot s^{-1}$ | $10^{-9}$ $m\cdot s^{-1}$ |

## Supplementary Discussion

### Mesh resolution dependence of fault zone evolution

In traditional rate and state dependent friction numerical modeling studies[1-6], the occurrence of slip instability depends on the choice of model grid size $\Delta x$ and characteristic slip distance, $\mathcal{L}$[4,7]. Our model is independent of $\mathcal{L}$, as the state of stresses is history dependent and replaces the equation for the state variable evolution in the rate and state formulation[8-12]. It has be shown that in models allowing for the localization of deformation with elastoplastic rheology, the fault width, $\Delta w$ is consistently ~3 times the grid size, $\Delta x$[13]. Since our model also includes this mechanism to simulate fault evolution through tectonic time scales, we did test the modeled fault zone width dependency on grid size. DES3D use Triangle[14,15] to generate unconstructed 2D triangle mesh. The triangles are not identical but similar. The model setup for this test is similar to the one shown in Figure 1a, which fault length is 90 km and the elastic plastic material in the shear zone has $a - b = -0.004$.

Test results are shown in Supplementary Table 2. Following theory, the fault zone width is about three times the typical shear zone grid size, $\Delta w = 3\Delta x$ (Supplementary Figure 4a). The average shear strain across a fault zone, $\boldsymbol{\varepsilon}_{\mathrm{xz}} = \Delta D/\Delta w$ is the ratio between the amount of slip, $\Delta D$ and the fault thickness, $\Delta w$. The shear strain rate is $\dot{\boldsymbol{\varepsilon}}_{\mathrm{xz}} = \Delta D/(\Delta w \cdot T_{\mathrm{L}})$, where $T_{\mathrm{L}}$ is the loading time or recurrence interval. The shear zone shear strain rate, $\dot{\boldsymbol{\varepsilon}}_{xz}$ and $\Delta D$ are constant following the numerical formulation and the model setup used in DES3D. Therefore $\Delta D/\dot{\boldsymbol{\varepsilon}}_{\mathrm{xz}} = \Delta w \cdot T_{\mathrm{L}}$ must remain constant. Since $\Delta w$ is proportional to $\Delta x$ ($\Delta w = 3\Delta x$) when the grid size $\Delta x$ is changed $T_{\mathrm{L}}$ must change accordingly. For a larger grid size, $T_{\mathrm{L}}$ must decrease and for a smaller grid size, $T_{\mathrm{L}}$ must increase. Test results in Supplementary Figure 4b shows that dependency is linear with a negative slope of -0.09. Accordingly the smaller the fault width (i.e. grid size) the longer the recurrence time, $T_{\mathrm{L}}$ which control the loading rate. We understand the dependency of our results as a function of grid size $\Delta x$. Given a fault width, $\Delta w$ and a recurrence interval, $T_{\mathrm{L}}$ from observations we can scale the grid size as to match the observations. However, we will include a natural length scale in our plastic formulation so as to get rid of this dependency using a regularization method[16].

**Supplementary Table 2.** Mesh resolution dependency.

| Resolution | X5 | X3 | X1 | X0.7 | X0.3 | X0.2 | X0.1 |
|---|---|---|---|---|---|---|---|
| Averaged Shear Zone Grid size | 450 m | 400 m | 350 m | 300 m | 200 m | 150 m | 90 m |
| Fault Thickness | 1100 m | 900 m | 700 m | 505 m | 350 m | 315 m | 210 m |
| Recurrence Rate | 280 year | 303 year | 309 year | 324 year | 346 year | 349 year | 365 year |

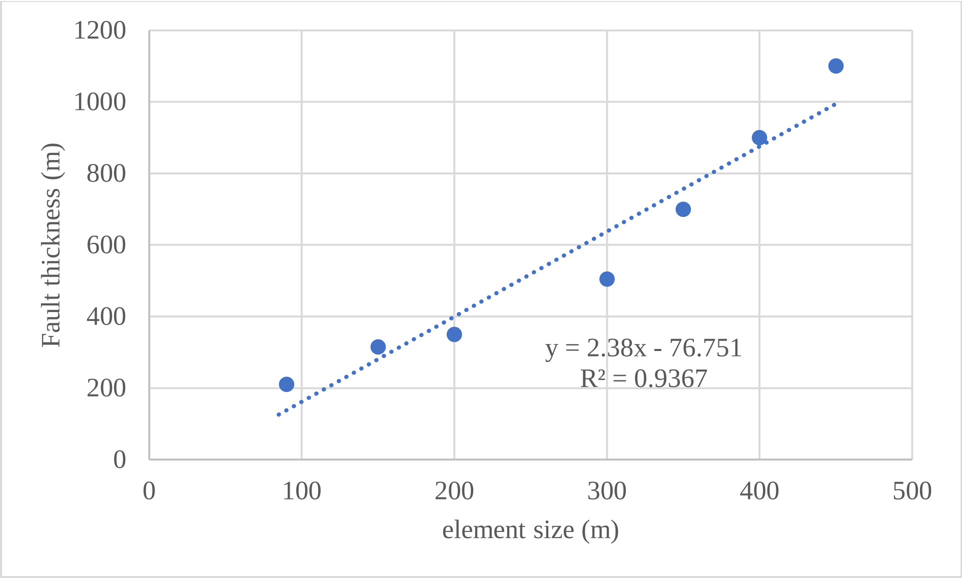


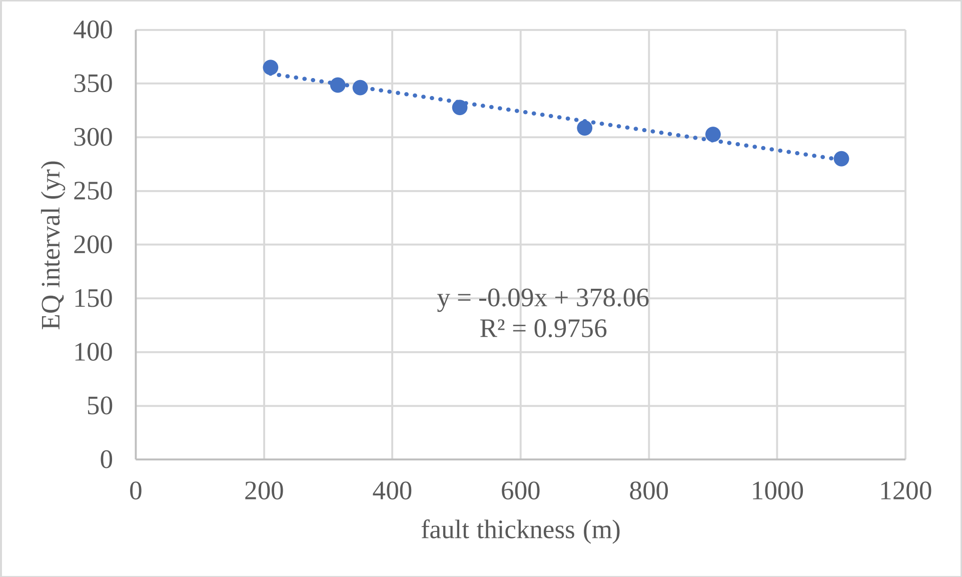


**Supplementary Figure 4.** a) Fault thickness dependency on grid size. b) Earthquake interval dependency on fault thickness (i.e. grid size).

**Supplementary References**


1 Colella, H. V., Dieterich, J. H. & Richards-Dinger, K. B. Multi-event simulations of slow slip events for a Cascadia-like subduction zone. *Geophysical Research Letters* **38**, (2011).

2 Liu, Y. J. Source scaling relations and along-strike segmentation of slow slip events in a 3-D subduction fault model. *J Geophys Res-Sol Ea* **119**, 6512-6533, (2014).

3 Liu, Y. J. & Rice, J. R. Aseismic slip transients emerge spontaneously in three-dimensional rate and state modeling of subduction earthquake sequences. *J Geophys Res-Sol Ea* **110**, (2005).

4 Liu, Y. J. & Rice, J. R. Spontaneous and triggered aseismic deformation transients in a subduction fault model. *J Geophys Res-Sol Ea* **112**, (2007).

5 Shibazaki, B. & Iio, Y. On the physical mechanism of silent slip events along the deeper part of the seismogenic zone. *Geophysical Research Letters* **30**, (2003).

6 Biemiller, J. & Lavier, L. Earthquake supercycles as part of a spectrum of normal fault slip styles. *J Geophys Res-Sol Ea* **122**, 3221-3240, (2017).

7 Lapusta, N. & Liu, Y. Three-dimensional boundary integral modeling of spontaneous earthquake sequences and aseismic slip. *J Geophys Res-Sol Ea* **114**, (2009).

8 Ruina, A. Slip Instability and State Variable Friction Laws. *Journal of Geophysical Research* **88**, 359-370, (1983).

9 Rice, J. R. Constitutive Relations for Fault Slip and Earthquake Instabilities. *Pure and Applied Geophysics* **121**, 443-475, (1983).

10 Dieterich, J. H. Modeling of Rock Friction .1. Experimental Results and Constitutive Equations. *Journal of Geophysical Research* **84**, 2161-2168, (1979).

11 Dieterich, J. H. Earthquake Nucleation on Faults with Rate-Dependent and State-Dependent Strength. *Tectonophysics* **211**, 115-134, (1992).

12 Dieterich, J. H. Constitutive properties of faults with simulated gouge. *Mechanical behavior of crustal rocks: the Handin volume*, 103-120 (1981).

13 Lavier, L. L., Buck, W. R. & Poliakov, A. N. B. Factors controlling normal fault offset in an ideal brittle layer. *J Geophys Res-Sol Ea* **105**, 23431-23442, (2000).

14 Shewchuk, J. R. Delaunay refinement algorithms for triangular mesh generation. *Comp Geom-Theor Appl* **22**, 21-74, (2002).

15 Shewchuk, J. R. Triangle: Engineering a 2D quality mesh generator and Delaunay triangulator. *Applied Computational Geometry Towards Geometric Engineering* **1148**, 203-222, (1996).

16 Zienkiewicz, O. C., Huang, M. S. & Pastor, M. Localization Problems in Plasticity Using Finite-Elements with Adaptive Remeshing. *Int J Numer Anal Met* **19**, 127-148, (1995).
